# Progress Toward Measles Elimination — Bangladesh, 2000–2016

**DOI:** 10.15585/mmwr.mm6628a3

**Published:** 2017-07-21

**Authors:** Sudhir Khanal, Rajendra Bohara, Stephen Chacko, Mohammad Sharifuzzaman, Mohammad Shamsuzzaman, James L. Goodson, Alya Dabbagh, Katrina Kretsinger, Deepak Dhongde, Jayantha Liyanage, Sunil Bahl, Arun Thapa

**Affiliations:** ^1^Immunization and Vaccine Development, World Health Organization (WHO) South-East Asia Regional Office, Delhi, India; ^2^Immunization Preventable Disease, Bangladesh Country Office, WHO; ^3^ Epidemiology and Surveillance, Directorate General of Health Services, Ministry of Health, Bangladesh; ^4^Global Immunization Division, Center for Global Health, CDC; ^5^Immunization and Vaccine Development, WHO Headquarters, Geneva, Switzerland.

In 2013, at the 66th session of the Regional Committee of the World Health Organization (WHO) South-East Asia Region (SEAR), a regional goal was established to eliminate measles and control rubella and congenital rubella syndrome* by 2020 (*1*). WHO-recommended measles elimination strategies in SEAR countries include 1) achieving and maintaining ≥95% coverage with 2 doses of measles-containing vaccine (MCV) in every district, delivered through the routine immunization program or through supplementary immunization activities (SIAs)^†^; 2) developing and sustaining a sensitive and timely measles case-based surveillance system that meets targets for recommended performance indicators; and 3) developing and maintaining an accredited measles laboratory network (*2*). In 2014, Bangladesh, one of 11 countries in SEAR, adopted a national goal for measles elimination by 2018 (*2*,*3*). This report describes progress and challenges toward measles elimination in Bangladesh during 2000–2016. Estimated coverage with the first MCV dose (MCV1) increased from 74% in 2000 to 94% in 2016. The second MCV dose (MCV2) was introduced in 2012, and MCV2 coverage increased from 35% in 2013 to 93% in 2016. During 2000–2016, approximately 108.9 million children received MCV during three nationwide SIAs conducted in phases. During 2000–2016, reported confirmed measles incidence decreased 82%, from 34.2 to 6.1 per million population. However, in 2016, 56% of districts did not meet the surveillance performance target of ≥2 discarded nonmeasles, nonrubella cases^§^ per 100,000 population. Additional measures that include increasing MCV1 and MCV2 coverage to ≥95% in all districts with additional strategies for hard-to-reach populations, increasing sensitivity of measles case-based surveillance, and ensuring timely transport of specimens to the national laboratory will help achieve measles elimination.

## Immunization Activities

In Bangladesh, MCV1, administered at age 9 months, was introduced nationwide^¶^ in 1989 (*4*), and MCV2, administered at age 15 months, in 2012. Administrative vaccination coverage** data are reported each year from the 64 districts in Bangladesh to the National Immunization Programme, where they are aggregated and reported to WHO and UNICEF through the Joint Reporting Form (JRF). WHO and UNICEF use reported administrative coverage and available survey results to generate annual estimates of vaccination coverage through routine services (*5*). As noted previously, in Bangladesh estimated coverage for MCV1 and MCV2 increased significantly (Figure). A routine vaccination coverage survey^††^ implemented in 2015 estimated 92% national coverage for MCV1 and 81% for MCV2.

**FIGURE:**
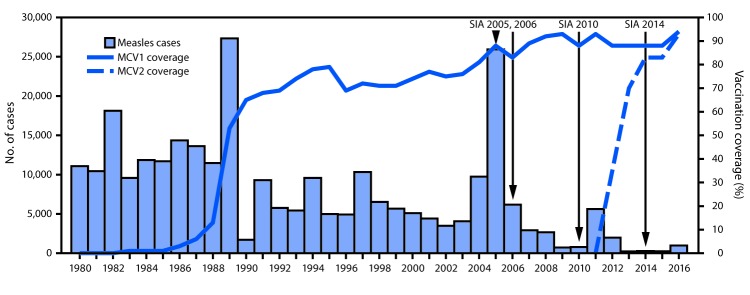
Aggregated measles cases,* estimated coverage**^†^** with the first and second dose of measles-containing vaccine (MCV1 and MCV2), and supplementary immunization activities (SIAs)**^§^****^,^****^¶^**^,^**^,^^††^ — Vaccine Preventable Disease Surveillance Report, Bangladesh, 1980–2016 * Laboratory-confirmed, epidemiologically linked and clinically compatible cases with fever; rash; and cough, coryza or conjunctivitis; reported as of Dec 2015 to World Health Organization (WHO) South-East Asia Region (2016 Joint Reporting Form [JRF]). ^†^ 1990–2015 coverage data from WHO/UNICEF estimates of national immunization coverage as published in July 2016; for 2016, coverage data are from country official estimates (submitted through 2016 JRF). ^§^ National measles catch-up SIA targeted children aged 9 mos–10 yrs, implemented in two phases: 1) Sep 2005, targeting 1,481,321 children; 2) Feb 2006, targeting 34,199,590 children. Overall administrative coverage >100%. ^¶^ National measles follow-up SIA targeted children aged 9–59 mos, conducted Feb 14–28, 2010, targeting 18,136,066 children. ** National measles-rubella catch-up SIA targeted children aged 9 mos–14 yrs, conducted during Jan 25–Feb 13, 2014 targeting 51,745,231 children. ^††^ Specific SIA dates are as follows: Sep 3–22, 2005; Feb 25–Mar 16, 2006; Feb 14–28, 2010; Feb 13–Mar 25, 2014.

During 2005–2006, a nationwide SIA using monovalent measles vaccine reached 36.0 million children aged 9 months–10 years (1.5 million during the first phase in 2015 and 34.2 million during the second phase in 2006) with >100% administrative coverage (*6*). A nationwide SIA in 2010 using monovalent measles vaccine reached 18.1 million children aged 9–59 months and achieved 100% administrative coverage. In 2014, a nationwide SIA using measles-rubella vaccine reached 53.6 million children aged 9 months–14 years and achieved >100% administrative coverage; 63 of 64 districts reported administrative coverage >95%. The post-SIA coverage survey estimated 90% vaccination coverage during the SIA.

## Surveillance Activities and Measles Incidence

In 2003, laboratory-supported case-based surveillance for suspected measles^§§^ was implemented in Bangladesh by adapting the existing acute flaccid paralysis surveillance system for polio detection; data are provided from 143 active and 625 passive surveillance sites in all 64 districts. In addition, aggregated measles cases^¶¶^ are reported by all health facilities through the National Health Management Information System and have been reported annually through the JRF since 2000. The difference in number of cases reported annually by these two parallel systems has decreased since 2013 (Table 1). Measles virus genotyping began in Bangladesh in 2014.

**TABLE 1:** Measles incidence,* number of reported measles cases by case classification, age group, and vaccination status — Vaccine Preventable Disease Surveillance Report, Bangladesh, 2001–2016

Year	WHO/UNICEF JRF aggregate reporting^†^		Measles case-based reporting^§^													
			No. of measles cases by classification					Age of confirmed measles cases, No. (%)					MCV doses received by confirmed measles cases, No. (%)			
	No. of reported measles cases	Incidence (cases/ million population)	Suspected^¶^	Confirmed**	Laboratory-confirmed	Epi-linked	Clinically compatible	<9 mos	9 mos–4 yrs	5–9 yrs	10–14 yrs	≥15 yrs	≥2	1	Zero	Unknown
**2000**	**5,098**	**40.0**	**—**	**—**	**—**	**—**	**—**	**—**	**—**	**—**	**—**	**—**	**—**	**—**	**—**	**—**
2001	4,414	34.2	—	—	—	—	—	—	—	—	—	—	—	—	—	—
2002	3,484	26.6	—	—	—	—	—	—	—	—	—	—	—	—	—	—
2003	4,067	30.6	721	640	56	584	—	46 (7.2)	303 (47.3)	212 (33.1)	59 (9.2)	20 (3.1)	—	169 (26.4)	436 (68.1)	35 (5.5)
2004	9,743	71.3	6,612	5,517	318	5,199	—	524 (9.5)	2662 (48.3)	1653 (30.0)	448 (8.1)	230 (4.2)	—	2,189 (39.7)	2,554 (46.3)	774 (14.0)
2005	25,934	186.4	27,539	14,877	739	14,138	—	1358 (9.1)	5670 (38.1)	4889 (32.9)	1763 (11.9)	1197 (8.1)	5 (0.0)	8,103 (54.5)	4,973 (33.4)	1,796 (12.1)
2006	6,192	43.7	7,820	3,058	169	2,889	—	306 (10.0)	1069 (34.9)	1076 (35.2)	392 (12.8)	215 (7.0)	1,085 (35.5)	1,146 (37.5)	773 (25.3)	54 (1.8)
2007	2,924	20.3	14,482	6	6	—	—	—	3 (50.0)	2 (33.3)	1 (16.7)	—	2 (33.3)	4 (66.7)	—	—
2008	2,660	18.1	8,308	139	16	123	5	12 (8.6)	77 (55.4)	33 (23.7)	7 (5.0)	10 (7.2)	2 (1.4)	68 (48.9)	69 (49.6)	—
2009	718	4.9	14,896	78	35	43	212	8 (10.3)	47 (60.3)	9 (11.5)	9 (11.5)	5 (6.4)	1 (1.3)	27 (34.6)	50 (64.1)	—
2010	788	5.3	14,745	66	51	15	440	7 (10.6)	24 (36.4)	19 (28.8)	3 (4.5)	13 (19.7)	27 (40.9)	17 (25.8)	22 (33.3)	—
2011	5,625	37.4	14,696	5,329	1,930	3,399	741	359 (6.7)	1820 (34.2)	1426 (26.8)	460 (8.6)	1264 (23.7)	1427 (26.8)	1,559 (29.3)	2343 (43.9)	—
2012	1,986	13.1	8,291	1,793	715	1,078	599	211 (11.8)	551 (30.7)	361 (20.1)	176 (9.8)	494 (27.6)	381 (21.2)	601 (33.5)	656 (36.6)	155 (8.6)
2013	237	1.5	5,229	200	77	123	325	32 (16.0)	70 (35.0)	45 (22.5)	14 (7.0)	39 (19.5)	28 (14.0)	88 (44.0)	63 (31.5)	21 (10.5)
2014	289	1.9	3,041	288	145	143	175	41 (14.2)	145 (50.3)	58 (20.1)	15 (5.2)	29 (10.1)	56 (19.4)	100 (34.7)	131 (45.5)	1 (0.3)
2015	240	1.5	3,435	250	158	92	64	32 (12.8)	125 (50.0)	42 (16.8)	22 (8.8)	29 (11.6)	87 (34.8)	81 (32.4)	75 (30.0)	7 (2.8)
2016	972	6.0	4,291	972	618	354	81	149 (15.3)	543 (55.9)	161 (16.6)	27 (2.8)	92 (9.5)	123 (12.7)	262 (27.0)	559 (57.5)	28 (2.9)

**Abbreviations**: epi = epidemiologically; JRF = Joint Reporting Form; MCV = measles-containing vaccine; WHO = World Health Organization.

* Measles incidence calculated based on reported confirmed measles cases and population by countries through WHO/UNICEF JRF.

^†^ National measles case data as reported to WHO South-East Asia Region Office (SEARO) as of December 2015 through the WHO/UNICEF JRF. Bangladesh uses administrative data reported through the national Health Management Information system (HMIS) to report in the JRF. The HMIS receives aggregated data from all the health facilities in the country, including private and public clinics and hospitals.

^§^ Data from case-based measles surveillance through the Vaccine Preventable Diseases surveillance network reported to WHO SEARO as of December 2016.

^¶^ An illness in any person a clinician suspects of having measles infection or in any person with fever and maculopapular rash and cough, coryza, or conjunctivitis.

** Includes laboratory-confirmed and epidemiologically linked cases. An epidemiologically linked case is one that meets the clinical case definition and is linked epidemiologically to a laboratory-confirmed or another epidemiologically confirmed case.

During 2013–2016, years for which data on key surveillance performance indicators*** (*7*) were available, the discarded nonmeasles, nonrubella rate increased nationally from 1.1 to 1.9 per 100,000 population; percentage of districts reporting at least two discarded nonmeasles, nonrubella cases per 100,000 population increased from 19% to 44%; percentage of suspected cases with adequate investigation initiated within 48 hours of notification increased from 87% to 94%; and the percentage of serology results reported by the laboratory within 4 days of specimen receipt increased from 82% to 94% (Table 2).

**TABLE 2:** National measles case-based surveillance performance indicator targets and progress toward meeting them — Vaccine Preventable Disease Surveillance Report, Bangladesh, 2013–2016

**Indicator**	**Target**	**Year**

2013	2014	2015	2016
Reporting rate of discarded nonmeasles cases at the national level per year	≥2	1.1	1.4	1.8	1.9
Percentage of subnational administrative units (districts) reporting at least two discarded nonmeasles cases per 100,000 population per year	≥80	19	18	36	44
Percentage of suspected measles* cases adequately investigated^†^ within 48 hours of notification	≥80	87	90	92	94
Percentage of suspected cases with adequate specimens^§^ tested for measles in a proficient laboratory^¶^	≥80	84	90	98	99
Percentage of results reported by the laboratory within 4 days of specimen receipt**	≥80	82	99	87	94
Percentage of weekly surveillance units reporting to the national level on time	≥80	85	89	91	97

* An illness in any person a clinician suspects of having measles infection, or in any person with fever and maculopapular rash and cough, coryza, or conjunctivitis.

^†^ Includes collection of all the following data elements about each suspected case of measles or rubella: patient name or identifiers, place of residence, place of infection (at least to district level), age (or date of birth), sex, date of rash onset, date of specimen collection, measles-rubella vaccination status, date of last measles-rubella or measles-mumps-rubella vaccination, date of notification, date of investigation, and travel history.

^§^ A blood specimen collected within 28 days of the onset of rash.

^¶^ A World Health Organization (WHO)-accredited laboratory that has an established quality assurance program or one with oversight by a WHO-accredited laboratory.

** Changed to 4 days from 7 day in 2015.

During 2000–2016, incidence of measles cases reported through the JRF decreased 84%, from 40.0 to 6.0 per million (Table 1). After implementation of nationwide measles SIAs in 2005–2006 and 2010, the number of confirmed measles cases decreased from 14,877 (2005) to 66 (2010), but increased to 5,329 in 2011, (Table 1). Following MCV2 introduction in 2012 and the nationwide measles-rubella catch-up SIA in 2014, confirmed measles cases declined from 1,793 in 2012 to 250 in 2015. In 2016, a program assessment was conducted using the WHO Programmatic Risk Assessment Tool for measles (*8*), and found eight districts at very high risk for measles transmission, 13 at high risk, 24 at medium risk, and 19 at low risk. In 2016, the number of confirmed measles cases increased, with 21 outbreaks in Sylhet Division and Cox’s Bazar District in the southeastern part of the country. Overall, 972 confirmed cases were reported. Outbreak response immunization activities targeting 100,000 children aged 9–59 months were conducted in two districts in December 2016. An outbreak investigation in affected areas revealed persistent low coverage with MCV1 and MCV2 through routine immunization (RI) and during the 2014 SIA. In addition, procedures for isolating measles cases were not followed, and nosocomial transmission of measles virus occurred in multiple health care facilities.

 Genotype results were available for two cases each in 2014 and 2015; all were genotype B3. No results were available for 2016.

## Discussion

During 2000–2016, after increasing MCV1 and MCV2 coverage and three SIAs, confirmed measles incidence in Bangladesh decreased 84% (*9*). In 2016, however, an outbreak occurred, and transmission has continued into 2017, revealing gaps in both RI and SIA coverage. The national vaccination coverage survey conducted in 2015 found the following most common reasons for a child being unvaccinated or partially vaccinated: 1) caretakers were too busy with other priorities, 2) caretakers did not remember to bring the child for vaccination, and 3) lack of information about when to bring the child for vaccination. These findings indicated the need for intensified social mobilization activities to strengthen RI, and a communication campaign is planned for 2017–2018.

In 2003, laboratory-supported measles case-based surveillance was implemented in Bangladesh by adapting the existing acute flaccid paralysis surveillance system for polio detection. Measles case-based surveillance indicators reflected underreporting and low sensitivity of the suspected measles case definition. Case-based surveillance sensitivity could be increased by expanding case-based surveillance reporting sites from acute flaccid paralysis reporting units to all health facilities in the country and by using the broad definition of “fever and maculopapular rash” (*10*). In addition, specimens for genotyping need to be collected from more chains of transmission to better track transmission pathways and identify outbreak sources. Infection prevention and control practices and isolation of measles cases in health care facilities needs strengthening to prevent nosocomial infection.

The findings in this report are subject to at least two limitations. First, administrative coverage resulted in overestimates of vaccination coverage through erroneous inclusion of SIA doses or doses administered to children outside the target age group, inaccurate estimates of the target population size, and inaccurate reports of the number of doses delivered. Second, surveillance data might substantially underestimate disease incidence, because not all patients seek care, and not all patients who seek care are reported.

The endorsement of the measles elimination goal in the comprehensive 2014–2018 plan for immunization in Bangladesh provides an opportunity to achieve and maintain measles elimination, by strengthening routine immunization services through innovative approaches, conducting high quality nationwide follow-up SIAs, enhancing case-based surveillance, and identifying opportunities for synergies with other public health programs. In 2015, the National Verification Committee for Measles Elimination was established, according to the global framework for the verification of progress toward measles elimination (*7*). As measles elimination nears, it will be necessary to conduct annual risk assessments and develop risk mitigation plans, conduct an immediate nationwide follow-up measles-rubella SIA to address current immunity gap among children aged 9–59 months, and build capacity for epidemiologic investigations and outbreak preparedness and response to rapidly identify and contain outbreaks.

**Summary**

**What is already known about this topic?**

Before 2000, estimated coverage with the routine first dose of measles-containing vaccine (MCV1) in Bangladesh was ≤75% nationally; no districts had ≥95% MCV1 coverage, and measles was a major cause of child death.

**What is added by this report?**

In 2014, a goal was set for measles elimination in Bangladesh by 2018. During 2000–2016, estimated MCV1 coverage increased from 74% to 94%. The routine second dose of measles-containing vaccine (MCV2) was introduced nationwide in 2012, and MCV2 coverage increased from 35% in 2013 to 93% in 2016. Approximately 108.9 million children were vaccinated during supplemental immunization activities (SIAs) in 2005–2006, 2010 and 2014. Annual reported measles incidence decreased 84%, from 40.0 to 6.0 per million population. Challenges to achieving elimination include low coverage with MCV1 and MCV2 and suboptimal performance of the measles case-based surveillance system.

**What are the implications for public health practice?**

Achieving ≥95% 2-dose measles vaccination coverage in all districts will require strengthening routine immunization services through innovative approaches and implementation of periodic high-quality SIAs. Improved measles case-based surveillance performance and increased surveillance sensitivity are needed for rapid case detection and outbreak preparedness and response.

## Footnotes

* Measles elimination is defined as the absence of endemic measles cases for a period of ≥12 months, in the presence of adequate surveillance. Rubella/Congenital Rubella Syndrome control is defined as 95% reduction in disease burden from 2008 levels.

† Supplementary Immunization Activities (SIAs) are immunization campaigns, typically carried out using two targeted age ranges. An initial, nationwide catch-up SIA targets all children aged 9 months–14 years, with the goal of eliminating measles susceptibility in the population. Periodic follow-up SIAs then target all children born since the last SIA and generally are conducted every 2–4 years and target children aged 9–59 months; the goal of a follow-up SIA is to eliminate any measles susceptibility that has accumulated in recent birth cohorts and to protect children who did not respond to the first dose of measles vaccine.

§ Suspected cases that have been investigated and discarded as a nonmeasles and nonrubella case using laboratory testing in a proficient laboratory or epidemiologic linkage to a laboratory-confirmed outbreak of another communicable disease that is neither measles nor rubella. It is estimated that the baseline rates of such discarded nonmeasles, nonrubella cases is two per 100,000 populations.

¶ The Expanded Programme on Immunization (EPI) began in 1979 as a pilot project and expanded in phases: the first phase (1985) included eight out of 490 thanas (a former administrative subdivision in urban areas of Bangladesh, equivalent to the current subdistrict [Upazila]); the second phase (1988) included 190 subdistricts. By the end of 1989 the EPI was expanded to the entire country.

** Administrative vaccination coverage is the number of vaccine doses administered divided by the estimated target population.

†† Bangladesh regularly conducts 30-cluster EPI immunization coverage surveys to evaluate routine and SIA coverage and is supported by partners. The last EPI coverage survey for which the results are available was conducted in 2015.

¶¶ National measles case data are reported to WHO South-East Asia Region Office) through the World Health Organization/UNICEF Joint Reporting Form (JRF) aggregate reporting annually. Bangladesh uses administrative data reported through the national Health Management Information system (HMIS) to report in the JRF. The HMIS receives aggregated data from all the health facilities in the country, including private and public clinics and hospitals.

*** Key surveillance performance indicators include 1) ≥2 discarded nonmeasles, nonrubella cases per 100,000 population at the national level per year; 2) ≥2 discarded nonmeasles, nonrubella cases per 100,000 per year in ≥80% of subnational administrative units; 3) an adequate investigation conducted within 48 hours of notification for ≥80% of suspected measles cases; 4) adequate specimens for detecting acute measles and rubella infection collected and tested in a proficient laboratory from ≥80% of suspected cases; 5) receipt of ≥80% of specimens at the laboratory within 5 days of collection; 6) laboratory reporting of ≥80% of serology results within 4 days of specimen receipt; and 7) on-time reporting of measles and rubella data to the national level by ≥80% of surveillance units.

## References

[1] World Health Organization Regional Office of South-East Asia. Resolution of the WHO Regional Committee for South Asia on measles elimination and rubella/congenital rubella syndrome control (SEA/RC66/R5). New Delhi, India: World Health Organization, Regional Office for South East Asia; 2013. http://www.searo.who.int/about/governing_bodies/regional_committee/rc66-r5.pdf?ua=1

[2] World Health Organization Regional Office of South-East Asia. Strategic plan for measles elimination and rubella and congenital rubella syndrome control in the South-East Asia Region—2014–2020. New Delhi, India: World Health Organization, Regional Office for South East Asia; 2014.http://www.searo.who.int/entity/immunization/documents/sear_mr_strategic_plan_2014_2020.pdf

[3] Government of People’s Republic of Bangladesh, Ministry of Health and Family Welfare. Comprehensive multi-year plan of the national immunization program of Bangladesh 2014–2018. Dhaka, Bangladesh: Government of People’s Republic of Bangladesh, Ministry of Health and Family Welfare; 2014.

[4] Talukdar LR, Basu RN, Shareef M, Khan MR, Nasiruddin NH. The near miracle: how immunization services are delivered in Bangladesh. In: Huq M, ed. Near miracle in Bangladesh. Dhaka, Bangladesh: Dhaka University Press Limited; 1991:57–74.

[5] World Health Organization; UNICEF. WHO/UNICEF estimates of national immunization coverage (WUENIC). Geneva, Switzerland: World Health Organization; New York, NY: UNICEF; 2015. http://www.who.int/immunization/monitoring_surveillance/data/en/

[6] World Health Organization. Supplementary immunization activities. Geneva, Switzerland: World Health Organization; 2017. http://www.who.int/immunization/monitoring_surveillance/data/Summary_Measles_SIA_Jan2000_Dec2017.xls?ua=1

[7] World Health Organization Regional Office of South-East Asia. Guidelines on verification of measles elimination and rubella/congenital rubella syndrome control in the WHO South-East Asia Region. New Delhi, India: World Health Organization Regional Office for South-East Asia; 2016. http://www.searo.who.int/entity/immunization/documents/mr_guidelines.pdf

[8] Lam E, Schluter WW, Masresha BG, Development of a district-level programmatic assessment tool for risk of measles virus transmission. Risk Anal 2015. Epub May 15, 2015. 10.1111/risa.12409 PMC10256314 25976980

[9] World Health Organization. Measles vaccines: WHO position paper. Wkly Epidemiol Rec 2009;84:349–60. 19714924

[10] Thapa A, Khanal S, Sharapov U, Progress toward measles elimination—South-East Asia Region, 2003–2013. MMWR Morb Mortal Wkly Rep 2015;64:613–7. 26068565 PMC4584924

